# Supplementary material for: Comparative multiomics analysis of cell physiological state after culture in a basket bioreactor
Source: Sci Rep. 2022 Nov 23;12:20161. doi: 10.1038/s41598-022-24687-4 (PMC9686226; doi:10.1038/s41598-022-24687-4)

W_023666039.1:1133846:1134187:-@NW_023666039.1:1133510:1134187:-@NW_023666039.1:1133510:1133779

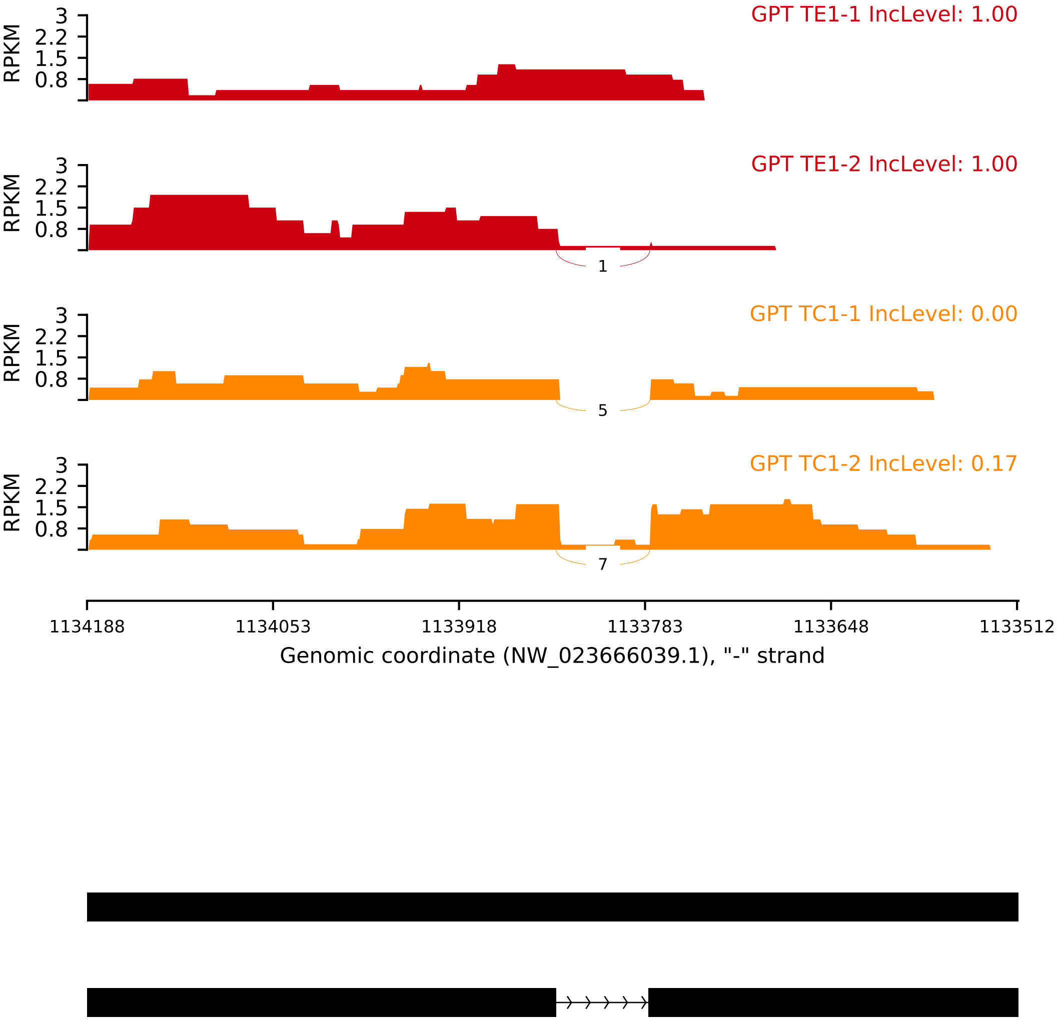

Supplement: Supplementary file 1 — Supplementary Information 1. [file 41598_2022_24687_MOESM1_ESM.zip › raw data/Transcriptomic raw data/7.AS/2.dasplot/TE1vsTC1/RI/2_GPT_NW_023666039.1_1133846_1134187_-@NW_023666039.1_1133510_1134187_-@NW_023666039.1_1133510_1133779_-.pdf]
